# The multiple meanings of global health governance: a call for conceptual clarity

**DOI:** 10.1186/1744-8603-10-28

**Published:** 2014-04-28

**Authors:** Kelley Lee, Adam Kamradt-Scott

**Affiliations:** 1 Faculty of Health Sciences, Simon Fraser University, Blusson Hall, 8888 University Drive, Burnaby, BC V5S 1S6, Canada; 2 Centre for International Security Studies, Department of Government and International Relations, University of Sydney, Room 384, H04 Merewether Building, Sydney, NSW 2006, Australia

**Keywords:** Global health governance, Global governance, Global health

## Abstract

**Background:**

The term global health governance (GHG) is now widely used, with over one thousand works published in the scholarly literature, almost all since 2002. Amid this rapid growth there is considerable variation in how the term is defined and applied, generating confusion as to the boundaries of the subject, the perceived problems in practice, and the goals to be achieved through institutional reform.

**Methodology:**

This paper is based on the results of a separate scoping study of peer reviewed GHG research from 1990 onwards which undertook keyword searches of public health and social science databases. Additional works, notably books, book chapters and scholarly articles, not currently indexed, were identified through Web of Science citation searches. After removing duplicates, book reviews, commentaries and editorials, we reviewed the remaining 250 scholarly works in terms of how the concept of GHG is applied. More specifically, we identify what is claimed as constituting GHG, how it is problematised, the institutional features of GHG, and what forms and functions are deemed ideal.

**Results:**

After examining the broader notion of global governance and increasingly ubiquitous term “global health”, the paper identifies three ontological variations in GHG scholarship - the scope of institutional arrangements, strengths and weaknesses of existing institutions, and the ideal form and function of GHG. This has produced three common, yet distinct, meanings of GHG that have emerged – globalisation and health governance, global governance and health, and governance for global health.

**Conclusions:**

There is a need to clarify ontological and definitional distinctions in GHG scholarship and practice, and be critically reflexive of their normative underpinnings. This will enable greater precision in describing existing institutional arrangements, as well as serve as a prerequisite for a fuller debate about the desired nature of GHG.

## Background

The term “global health governance” (GHG) is now widely used within research, policy and practice. Since the introduction of the concept more than a decade ago [1], over one thousand scholarly works have been published on the subject across a wide range of disciplines. Amid this rapid growth in scholarship, however, there has been considerable variation in how the term has been defined and applied, generating confusion as to the boundaries of GHG as a subject, the perceived problems in practice, and the goals to be achieved through institutional reform. An important review of the GHG literature by Ng and Ruger [2] identifies a transition from international health governance (IHG) to global health governance, major actors, and challenges and successes in GHG. The authors also analyse the framing of health as national security, human security, human rights, and global public good, and the implications of these various frames. While this review usefully describes the emerging boundaries of scholarship on GHG, the different ways in which GHG is defined has not been examined to date.

This paper seeks greater conceptual clarity through a critical review of the scholarly literature on GHG to date. After a brief discussion of the broader notion of global governance, and the increasingly ubiquitous term “global health”, the paper identifies three ontological variations in the literature. It then interrogates three common meanings of GHG that have emerged – globalisation and health governance, global governance and health, and governance for global health. It is argued that there is a need to separate out their distinct meanings and, in particular, to be critically reflexive of their normative underpinnings. In doing so, the paper demonstrates the need for greater precision in the use of these terms, as well as a more critical approach to GHG scholarship, as a prerequisite for a fuller debate about the desired nature of GHG.

## Methods

A separate scoping study by Catherine Jones and Kelley Lee on methodological approaches used in GHG research was used to inform this analysis. The scoping study conducted a keyword search of public health, law and social science databases (Medline, Global Health, Cinahl Plus, Political Science Complete, International political science Abstracts, International Bibliography of the Social Sciences, PAIS International, Sociological Abstracts, Worldwide Political Abstracts, FRANCIS) to identify peer-reviewed articles on GHG published from 1990 onwards. Additional works, notably books, book chapters and scholarly articles not currently indexed (such as the journal *Global Health Governance*), were identified through Web of Science citation searches.

Given variation in terms used in the GHG literature, Boolean tools of adjacency or proximity searches were used to retrieve relevant results, as well as a truncation of the term governance (governa* and a list of Boolean OR operators to ensure inclusion of govern, governs, governed, and governing), but to the exclusion of government, governments, governmental). The searches for these terms were conducted primarily on abstract and title searchers (AB and TI), and then expanded to full text searches as a search mode option, when available. Full text searches (includes a search of title, abstract, keywords and full text of the article) were conducted primarily in the social science databases. The above search initially yielded 1773 articles through the database searches and 127 additional works. Duplicate citations (n=755) and book reviews (n=69) were removed, leaving 1076 published works. Commentaries, editorials and brief reports were then removed, leaving a total of 250 scholarly works on GHG published between January 1990 and December 2013. For the purpose of this paper, to critically review how the concept of GHG is applied in the existing peer-reviewed literature, the authors examined the remaining works in terms of what they claim constitutes GHG. More specifically, we describe what are considered the institutional features of GHG, how it is problematised, and what forms and functions are deemed ideal. Based on this analysis, we identify three distinct definitions of GHG.

## Results

### What is “global governance”?

For conceptual clarity, it is useful to review the conceptual origins of GHG, a term which began to be used in the 1990s amid angst about the shortcomings of international health cooperation and the growing impacts of globalization on health determinants and outcomes [3]. The term, coined by Dodgson et al. [1] draws from the broader concept of *global governance*, an already established subject area within International Relations (IR). In broad terms, *governance* is needed when a collective of individuals comes together to accomplish an agreed end. The resultant arrangement constitutes governance if there is recognised authority, agreed rules for decision making, and accountability. Governance through governments (state-based institutions with formal authority and powers to manage and regulate social actors) is familiar. Governance may constitute institutional arrangements involving both state and non-state actors.

When governance extends beyond one country, arrangements may involve state actors (e.g. World Health Organisation), non-state actors (e.g. International Federation of Pharmaceutical Manufacturers) or both (e.g. Global Fund to Fight HIV/AIDS, Malaria and Tuberculosis). Particular challenges arise for governance beyond the state due to what International Relations scholars call “anarchy” (the absence of overarching political authority above sovereign states). At the same time, the end of the Cold War and the acceleration of globalisation during the late twentieth century have increased calls for effective collective action to address shared challenges such as climate change, disease outbreaks and illicit activities [4]. Non-state actors have also become significantly more prominent, arguably rivalling and even eroding the capacity of states to govern. The perceived need for global governance, therefore, has coincided with shifting power between state and non-state actors. This dilemma has produced a flurry of global governance scholarship grappling with the challenge of creating institutions that effectively “govern without government” in a global era [5].

Since the early 1990s, global governance scholarship has been characterised by rich theoretical argument and empirical analysis, but also imprecision and contestation in meaning. Global governance has been variably described as an objective, a process and an ideal [6-8]. For example, the United Nations Commission on Global Governance [9] defines the concept as

the sum of the many ways individuals and institutions, public and private, manage their common affairs. It is a continuing process through which conflicting or diverse interests may be accommodated and cooperative action may be taken. It includes formal institutions and regimes empowered to enforce compliance, as well as informal arrangements that people and institutions either have agreed to or perceive to be in their interest.

Young [10] refers to “the combined efforts of international and transnational regimes”, while Whitman [11] is more expansive:

governance can be understood as global by the way of the nature and sum of many governance systems, across sectors and at levels high and low. In the sense that all the world’s human systems can be regarded as interconnected, however patchy and incoherent in their entirety, ‘global governance’ is an established fact of the human condition.

Noting criticisms this might embrace “virtually anything”, Finklestein [12] suggests global governance is “governing, without sovereign authority, relationships that transcend national frontiers. Global Governance is doing internationally what governments do at home”.

Despite a lack of definitional consensus, common threads emerge. One agreed feature is that global *governance* does not equate to, nor is it considered synonymous with, global *government.* As Rosenau [13] argues:

Both refer to purposive behaviour, to goal-orientated activities, to systems of rule; but government suggests activities that are backed by formal authority, by police powers to insure the implementation of duly constituted policies, whereas governance refers to activities backed by shared goals that may or may not derive from legal and formally prescribed responsibilities and that do not necessarily rely on police powers to overcome defiance and attain compliance.

To speak of global governance, however, where no supranational authority exists can be confusing. What is meant by “governance without government”? Gordenker and Weiss [14] address this uncertainty by defining global governance as

efforts to bring more orderly and reliable responses to social and political issues that go beyond capacities of states to address individually. Like the NGO universe, global governance implies an absence of central authority, and the need for collaboration or cooperation among governments and others who seek to encourage common practices and goals in addressing global issues.

In this regard, authors such as Biermann et al. [15] have attempted to refine the locus of investigation by focusing on “governance architectures”, defined as “the overarching system of public and private institutions, principles, norms, regulations, decision-making procedures and organizations that are valid or active in a given issue area of world politics”.

A second characteristic that resonates throughout the global governance literature is the importance of non-state actors. As Krahmann [7] summarises, “global governance is characterized by the need for greater collaboration among governments and nongovernmental actors as the result of states being faced with new and growing demands on the one hand and shrinking resources on the other”. In general, there is an expectation for governments to engage more closely with non-state actors as a means of strengthening representation, accountability and transparency [16-20]. For instance, Castells [21] argues that “the relationship between the state and civil society is the cornerstone of democracy”.

A third theme in the global governance literature is the search for innovative institutional arrangements to adapt to, and manage, the externalities arising from increased transboundary flows [22,23]. The need to reform decision-making fora and processes [21,24], representative of the transboundary nature of political constituencies and their interests, has been important debates in this literature. Since 2008, the global financial crisis has generated attention to strengthen global economic governance [17,25,26].

A fourth theme is that global governance is not limited to the global level but embraces the local to the supranational. Conceptualised in this way, as Betsill and Bulkeley [8] write, “[t]he development of a governance perspective involves recognising the roles of supranational and subnational state and nonstate actors, and the complex interactions between them, in the process of governing”. A key advantage of such a dynamic, it is argued, is that “power is seen to accumulate from multiple sources of authority, including expertise and moral positions, and to be a relational concept” [8]. A downside is the potential for governance systems to become overly complex, with many “voices” claiming the right to be heard across different fora. Beyond state authority, therefore, the challenge is not only achieving consensus, but determining legitimate enfranchisement. In a world of multiple sources of authority, whose voices should prevail in global governance?

An important cross cutting theme in the literature is the achievement of “good” global governance [27,28]. The normative basis of what constitutes good governance has been the subject of much scholarly and policy debate. Applied to the public sector of developing country governments, the UN Development Programme (UNDP) frames good governance principles within the context of sustainable human development and poverty alleviation [29]. In contrast, the World Bank has focused on creating efficient and effective public administration that cast democratic governments in the role of, inter alia, enabling markets to thrive as a core component of economic development [30]. Despite continued contestation surrounding different interpretations of the purpose and meaning of good governance it has remained a core expectation of donors for aid recipient countries [6]. Over time, indicators of good governance have become more broadly defined to embrace, inter alia, principles of legitimacy, strategic vision, effectiveness, accountability and transparency, and the rule of law [31].

Overall, understanding the substantial literature on global governance is useful for clarifying the boundaries of GHG scholarship. The distinction by Dingwerth and Pattberg [32], between global governance as an analytical concept and normative perspective, is especially relevant in this respect. On global governance they write:

Besides its use as an analytical concept that attempts to capture the - actual, perceived, or constructed - reality of contemporary world politics…the concept is often used to denote a specific political program, expressing either a normative perspective on how political institutions should react to the reduced steering capacity of national political systems or a critical perspective that refers to global governance as a hegemonic discourse.

### What is “global health”?

As Koplan et al. [33] describe, “global health is fashionable”, as expressed in an explosion of initiatives since the late 1990s. Many governments identify global health as a key foreign policy objective [34-36]. Philanthropies, international organisations, NGOs, private businesses and public-private partnerships have identified global health as a priority [37,38]. Academic institutions have created new, or renamed existing, teaching and research programmes [39]. All of this has been accompanied by significant spending on global health activities [40].

Like global governance, however, there are diverse definitions of global health which, alongside its prevalent use, risks rendering it meaningless. Definitions abound but most lack conceptual rigour. Brown et al. [41] locate the historical roots of international health in nineteenth century European imperialism when the priority for collective health action was controlling epidemic diseases spreading from colonised territories. In contrast, global health encompasses a shift to thinking about “the health needs of the people of the whole planet above the concerns of particular nations”. Koplan et al. [33] distinguish public (or population), international (interstate) and global health, with the latter variably “thought of as a notion (the current state of global health), an objective (a world of healthy people, a condition of global health), or a mix of scholarship, research, and practice (with many questions, issues, skills, and competencies)”.

An alternative approach is to focus on the nature or characteristics of a health issue to categorise it as a “global health” problem requiring collective action. The issue must be caused by factors, or possess the capability to occur and/or spread in ways, that transcend territorial geography – to be “transboundary” – to be classified as a global health problem. Needless to say, not every health problem qualifies. Guinea worm, for example, remains a residual health threat in many parts of Africa. By comparison, significant political and financial capital has been expended on HIV/AIDS. The distinction drawn might be explained, at least in part, by the fact that the former is transmitted via an animal vector only prevalent in certain parts of the world. The other is a disease spread by close contact with infected bodily fluids, an event that can occur in any part of the world. In this same sense, influenza (both seasonal and pandemic) is considered global due to its transboundary transmissibility, while malaria could be classified as not a global health issue because it is limited to tropical climates. Focusing on health determinants, lung cancer or liver cirrhosis could be described as global because their causal factors are transboundary (i.e. transnational tobacco and alcohol companies). The nutritional taboos practiced in some cultures, leading to nutritional deficiencies in pregnant women and children, is a serious problem but not a global health issue by virtue of its localised practice.

Overall, the term global health has been confused by its variable use both descriptively and prescriptively. Descriptively, global health is used to describe certain health issues (e.g. epidemics, neglected diseases), or health needs within certain geographies (e.g. low-income countries) or populations (e.g. poor, HIV-infected). Prescriptively, global health is used aspirationally to advocate for certain health goals (e.g. universal health coverage, global health security) for selected geographies or populations. Importantly, as we argue elsewhere, both descriptive and prescriptive uses of the term are embedded within particular normative frameworks or world views which shape thinking and practice about what is the “problem” in global health and what are the legitimate “solutions” [42]. In other words, the theory and practice of global health must be critically understood within the context of shifting material and ideational circumstances. This social construction of the term has had direct implications for the “practice” of global health and how, in turn, it impacts on material reality [43,44].

## Ontological variations in GHG scholarship

It is within the above context that fuller understanding of the conceptualisation of GHG to date can be located. Emerging from a longstanding literature on international health cooperation [45], a series of discussion papers, commissioned by WHO in the late 1990s, set out the parameters of the emerging subject of GHG [1,46-49]. In the substantial literature published over the past decade and a half, three core ontological variations can be observed. First, the scope of institutional arrangements deemed to fall under the rubric of GHG varies substantially. Some focus on issue-specific agreements to protect and promote population health such as the Framework Convention on Tobacco Control (FCTC) [50-52], International Health Regulations (IHR) [53,54] or Doha Declaration on TRIPS (Agreement on Trade Related Intellectual Property Rights) and Public Health [55,56]. Others take a broader approach, seeking to understand the increasingly crowded and complex institutional arrangements shaping global health policy including the rise of new state [57-61] and non-state actors [62-71], and their assemblage into public-private partnerships [72-75]. Still others consider institutions beyond the health sector, such as the World Bank, International Monetary Fund (IMF) and World Trade Organisation (WTO), which have broader impacts on the social determinants of health [76-79].

A second variation concerns the perceived strengths and weaknesses of existing institutional arrangements known as GHG. There is general recognition of the need for collective action on shared health challenges, especially given the exigencies of globalisation, and consensus that existing institutional arrangements currently fall short. There are different perceptions, however, about the nature of these shortfalls. Some scholars call for mechanisms to generate and apply new knowledge or technical interventions [80-82]. Others perceive the deficiencies of GHG as organisational, with analyses focused on improving administrative, management or legal structures and processes [83-87]. Where there are reports of poor standards of practice, efforts are given to establishing and enforcing quality control measures [88]. A further group see GHG shortcomings as stemming from resource deficiencies, and thus focus on the creation of new and innovative funding mechanisms, incentive systems or capacity building [89-92]. A critical group of scholars see systemic inequities in the institutional arrangements presently governing global health as the underlying problem, with the politically and economically powerful disproportionately shaping and benefiting [38,93-97]. What is “right” or “wrong” about GHG, in other words, has elicited diverse starting points.

A third, and perhaps most fundamental, variation concerns the ideal form and function of GHG. Dodgson et al. [1] broadly define GHG as the rules and procedures by which collective action is taken to achieve agreed goals that protect and promote health within a global context. However, what those goals should be, and the institutional arrangements deemed necessary to define, prioritise and pursue such goals, are invariably shaped by normative frameworks. In some cases, these frameworks are explicitly stated [98-102]. In much of the existing literature, norms are embedded within prescriptive arguments for institutional arrangements regarding the exercise of authority, means of representation, distribution of resources, and systems of accountability and transparency. The emergent nature of GHG complicates such deliberations, with contested normative frameworks shaping aspirations for GHG [41,103]. Overall, these ontological variations in the perceived scope, strengths and weaknesses, and ideal form and function of GHG define a fast growing, but conceptually and empirically incoherent literature. As Berridge et al. [47] conclude, “global health and global health governance are ‘slippery’ concepts which have eluded attempts to define them with any degree of clarity”.

### Three concepts of global health governance

The ontological variations described above explain, in large part, differences in how GHG has been conceptualised to date. This paper identifies three distinct uses of the term GHG: (a) globalisation and health governance; (b) global governance and health; and (c) governance for global health. It is argued that each of these distinct concepts, often used interchangeably within the existing literature, derive from particular normative frameworks which, in turn, shape their conceptualisation of the scope and purpose of GHG. The application of these distinct concepts is often unreflexive, resulting in a lack of conceptual clarity in this rapidly emerging subject area.

#### Globalisation and health governance

A substantial proportion of the GHG literature refer to the institutional actors, arrangements and policy making processes that govern health issues in an increasingly globalised world. Setting aside variations in the definition of “global health” described above, which differ in what qualifies as *global* among health issues, this concept of GHG is primarily concerned with the health-related institutions that govern collective responses to such issues [80,104]. Hein et al. [105] exemplify this understanding of GHG:

Contemporary GHG is characterized by a polycentric, distributed structure and a substantive concern with issues that affect populations worldwide directly (for example, the global spread of infectious diseases or antibiotic resistance) or indirectly (for example, political instability and global insecurity arising from extreme socio-economic inequality). Global health governance now requires management of not merely of specific transborder epidemics, like SARS or avian influenza, but of the host of issues in health that arise at the intersection of a globalized economy and lives lived in particular localities.

This concept of GHG has emerged directly from concerns about the inner workings, and external relationships, of the WHO. WHO is referred to as a GHG institution [78,106,107], holding constitutional authority within the UN system to adopt agreements, issue guidelines and provide technical assistance to member states to protect and promote health. However, the limitations in the organisation’s capacity to fulfil this role, attributed to resource constraints, bureaucratic complexities, political machinations or obsolescence as a state-focused institution within a globalising polity, has led to substantial reflection about WHO alongside other health-relevant UN bodies, global public-private partnerships for health, charitable foundations, civil society organisations and corporations [73,108]. Attention has focused on reforming existing institutional arrangements so that they are more effective, foremost, but also more representative, transparent and accountable [62,63,75,109-112].

There has been a strong focus, in particular, on institutional innovation arising from the need to govern beyond governments [86,87,113-116]. Fidler [117], for example, defines GHG as “the use of formal and informal institutions, rules, and processes by states, intergovernmental organizations, and nonstate actors to deal with challenges to health that require cross-border collective action to address effectively”. Harman [78] suggests that GHG “refers to trans-border agreements or initiatives between states and/or non-state actors to the control of public health and infectious disease and the protection of people from health risks or threats”. Davies [107] seeks to differentiate between *statist* and *globalist* perspectives, noting that the former views GHG as a tool for state-centric governance, whereas the latter sees GHG as “a new form of politics that transcends state sovereignty and directs the focus on individuals and their vulnerabilities”.

#### Global governance and health

A second way GHG has been conceptualised in the literature has been to describe how global governance institutions outside of the health sector have influenced the broad social determinants of health. This literature initially dealt with the policy decisions of multilateral financial institutions, notably the World Bank and IMF, along with other institutional players in global economic relations such as the WTO and OECD [118]. The health impacts in low- and middle-income countries of the Structural Adjustment Programmes (SAPs) of the World Bank, for instance, elicited much interest [93,119-121]. The IMF has received similar attention [78,119]. Since the mid 2000s, increasing attention has been paid to the G8 [77,94,122,123].

The proliferation of trade and investment agreements since the 1990s has also received substantial attention in the GHG literature. To date, this has largely focused on the impact of the Agreement on Trade Related Intellectual Property Rights (TRIPS) on access to medicines [71,124]. The stalling of the Doha Round of WTO negotiations from 1999 has seen a forum shift to the regional and bilateral levels, making it even more difficult for health goals to be represented. The dispute settlement process of bilateral investment agreements (BITs) has raised particular concerns because they give corporations legal standing to bring disputes against governments for social and environmental policies that may impact on their economic interests.

Much of this literature is also critical of the pro-market orientation of these institutions which, it is held, conflicts with core principles in public health such as equity and social justice [79]. Indeed, critics argue that these institutions do not recognise, or chose to ignore, the normative basis of their policies [125,126]. As well as challenging neoliberal assumptions that better wealth will eventually “trickle down” and lead to better health, as ideologically-driven and structurally flawed [93], this literature has focused on improving good governance within these institutions. How global governance institutions are governed themselves, and how they implement their decisions at the national level has received growing attention in the GHG literature.

#### Governance for global health

A third use of GHG concerns what governance arrangements are needed to further agreed global health goals. This use of the term is more normative, not only responding to the impacts on health of a globalising world, but seeking to achieve particular goals such as access to medicines, health equity or primary health care [127], or principles such as human rights [68,128,129] and social justice [130]. As discussed above, different definitions of global health abound. In this context, global health is defined in terms of the poor, vulnerable and disadvantaged, most often to refer to the health needs of the developing world. Innovation in institutional design is again advocated, but for the purpose of achieving a specific end, rather than to improve health governance more generally. For example, Pogge [90] argues for a health impact fund to enhance justice and efficiency in global health. Atun and Kazatchkine [131] discuss the GFTAM’s experience of promoting country ownership and stewardship of health programmes. Gostin [132] and others [133] call for a Framework Convention on Global Health, launched under the auspices of the Joint Action and Learning Initiative on National and Global Responsibilities for Health (JALI):

With global health justice as a core principle, JALI will enable and prioritize input of the people who suffer most from today’s national and global health inequities - marginalized communities, people who live in extreme poverty, women, persons with disabilities, and other disadvantaged populations.

As well as playing a prescriptive role, norms and values in GHG has become the subject of growing scholarship. For example, Ruger [134] interrogates the ethical foundations of GHG while Stewart et al. [135] and Brown [130] examine the importance of values. The application of social constructivist approaches to understanding GHG [42-44], focused on the influence of ideational power in shaping thinking and action, is an important advance towards greater reflexivity.

## Conclusion

The growth in scholarly and policy attention to the challenges of achieving collective action to address shared health needs in a globalising world is an important development. The aim of this paper, to interrogate the conceptualisation of GHG to date, reveals a rich but siloed literature. Conceptual imprecision continues to abound, largely due to the ongoing vagaries of the term global health. As Koplan et al. [33] argue, defining what we mean by global health is critical to “agreement about what we are trying to achieve, the approaches we must take, the skills that are needed, and the ways that we should use resources”. The GHG literature to date is also multidisciplinary, broadly stemming from either the practically-oriented public health community, or the more theoretically-oriented social science community. Both communities also embrace diverse perspectives. A few writers bridge this disciplinary divide [38,42,46,49,135], but the literature suggests that the theory and practice of GHG are rarely brought together. Moreover, the subject is separated by a focus on specific issue areas, population groups, geographies, institutional players and normative frameworks.

Recognition of the conceptual roots of the term GHG in global governance and global health are important starting points for advancing the field. This paper offers an initial mapping of the critical mass of literature on GHG now available. This leads to recognition of the ontological variations that have emerged and, in turn, the different uses of the concept. The normative frameworks embedded within these works, and their aspirational goals, are also important to acknowledge. Above all, this paper concludes that more critical reflection on how GHG is conceptualised is a prerequisite for fuller debate about efforts to improve its practice.

## Competing interests

The authors declare that they have no competing interest.

## Authors’ contributions

KL and AKS participated in the design of the study. KL and AKS drafted and revised the paper, and both authors approved the final manuscript.
